# Rational Design of Porous Covalent Triazine-Based Framework Composites as Advanced Organic Lithium-Ion Battery Cathodes

**DOI:** 10.3390/ma11060937

**Published:** 2018-06-02

**Authors:** Ruoxin Yuan, Wenbin Kang, Chuhong Zhang

**Affiliations:** State Key Laboratory of Polymer Materials Engineering, Polymer Research Institute of Sichuan University, Chengdu 610065, China; 2015223090029@stu.scu.edu.cn (R.Y.); wenbin.kang@scu.edu.cn (W.K.)

**Keywords:** covalent triazine-based frameworks, carbon materials, graphene, lithium-ion batteries

## Abstract

In an effort to explore the use of organic high-performance lithium ion battery cathodes as an alternative to resolve the current bottleneck hampering the development of their inorganic counterparts, a rational strategy focusing on the optimal composition of covalent triazine-based frameworks (CTFs) with carbon-based materials of varied dimensionalities is delineated. Two-dimensional reduced graphene oxide (rGO) with a compatible structural conformation with the layered CTF is the most suitable scaffold for the tailored mesopores in the polymeric framework, providing outstanding energy storage ability. Through facile ionothermal synthesis and structure engineering, the obtained CTF-rGO composite possesses a high specific surface area of 1357.27 m^2^/g, and when used as a lithium ion battery cathode it delivers a large capacity of 235 mAh/g in 80 cycles at 0.1 A/g along with a stable capacity of 127 mAh/g over 2500 cycles at 5 A/g. The composite with modified pore structure shows drastically improved performance compared to a pristine CTF, especially at large discharge currents. The CTF-rGO composite with excellent capacity, stability, and rate performance shows great promise as an emerging high-performance cathode that could revolutionize the conventional lithium-ion battery industry.

## 1. Introduction

As the most widely utilized member in the secondary battery family, the lithium-ion battery (LIB) has been seen in numerous applications across a diverse spectrum, ranging from use in portable consumer electronics like cellphones, cameras, and personal computers etc. to applications with greater power/energy consumption like the grid-scale residential electricity supply [1]. Meanwhile, due the intensifying demand for a clean energy supply, LIBs are becoming popular, and their use is gradually superseding that of traditional internal combustion engines due to their ability to store intermittent clean energy from solar cells [2,3,4], nanogenerators [5,6], and thermolelectrics [7,8] etc. as chemical energy and reversibly convert it to electricity in times of need. Since the introduction to the market of LIBs in 1991 by Sony [9], great advances have been made towards the realization of electrochemically active electrodes with high capacity and a long cycling life. Although there has been commercial success with many viable applications, the scientific community still strives to explore the next generation of high-performance electrodes for use in highly demanding industries like that of electric vehicles. It seems the cathode industry in particular is not progressing, and sluggish development is seen in conventional inorganic intercalation-type ielectrodes because of low theoretical capacity, limited metal element deposits, and high costs [10].

On the other hand, nanoporous organic polymer networks, such as hyper-cross-linked polymers (HCPs) [11], covalent organic frameworks (COFs) [12], and conjugated microporous frameworks (CMPs) [13] etc., characterized by interconnected pores and exceptionally large surface areas [14], are receiving escalating attention due to their applications in gas adsorption [15], catalysis [16], and energy storage [10,17,18,19], etc. Notably, covalent triazine frameworks, which are known as CTFs [20,21,22,23], have been investigated and a unique n, p doping mechanism that enables lithium ion storage makes them prospective high-performance cathodes for LIBs [10,24,25,26]; their high surface area has also prompted wide applications in other electrochemical energy storage devices [27,28,29,30]. However, the low intrinsic conductivity and ultra-small micropores that hamper facile counter-ion diffusion lead to unsatisfactory energy storage performance, and these issues should be immediately addressed in order to maximize their great energy storage potential.

Carbon materials that exist in versatile forms with excellent conductivity represent promising scaffolds on which hybrid composites with tailored structures and properties can be obtained [31,32]. Herein, a study of the composition of the CTF and carbon materials of different dimensionalities is conducted with the aim of modifying the original monodisperse microporous framework and simultaneously improving its conductivity. The growth compatibility of the CTF with carbon materials is analyzed and reduced graphene oxide (rGO) is determined to be the most suitable scaffold for the development of a hierarchical pore structure that boosts its energy storage performance as an LIB cathode. The CTF-rGO composite manages to deliver a large reversible capacity of 235 mAh/g in 80 cycles at 0.1 A/g and maintains a capacity of 125 mAh/g after 1000 cycles at 2 A/g. The greatly improved performance compared to an unmodified pristine CTF provides evidence of the effectiveness of rational structure engineering for the realization of superior electrochemical performance. 

## 2. Materials and Methods 

### 2.1. Chemicals

Zinc chloride (ZnCl_2_, ACS), terephthalonitrile (98%) and *N*-methylpyrrolidone (NMP, 99.9%) were purchased from Aladdin Reagent (Shanghai, China) and used without further purification. Graphite oxide (GO) was purchased from The Sixth Element Materials Technology Co., Ltd (Changzhou, China). Carbon nanotubes (CNTs) (TNIM4, 10~30 nm in diameter, 10~30 μm in length) were purchased from Chengdu Organic Chemicals Co. Ltd. (Chengdu, China). Hydrochloric acid (HCl), methanol (MeOH), tetrahydrofuran (THF), and chloroform (CHCl_3_) were purchased from Chengdu Kelong chemicals (Chengdu, China). 

### 2.2. Synthesis of CTF and the Composites

Carbon spheres (CS) were synthesized according to the literature [33]. Reduced graphene oxide (rGO) was prepared by ultra-sonication of graphite oxide powder in water followed by hydrazine hydrate reduction [34]. Graphene aerogel (GA) was prepared by a simple hydrothermal method [35]. In a typical synthesis procedure of a CTF composite, a 300-mg monomer (terephthalonitrile), 10 mol equivalent of ZnCl_2_, and 30 mg of carbon material were ground thoroughly in a mortar in an Argon-filled glove box. The mixture was then transferred into a quartz tube (1 cm in diameter, 20 cm in length), evacuated, sealed, and heated to the desired temperature. The tube was then cooled down and opened. The black monolithic product was grounded thoroughly and subsequently washed in diluted HCl for 72 h. Soxhlet extraction with CHCl_3_, THF, and MeOH was then conducted on the obtained samples in 12 h before drying in vacuum at 120 °C overnight. 

### 2.3. Characterizations

FT-IR measurements were carried out using a Nicolet iS50 apparatus (Thermo Scientific, Waltham, MA, USA). Powder X-ray diffraction (XRD) measurements were performed on a Rigaku Smart Lab (3) with Cu Ka. radiation. The morphology of the polymeric frameworks was analyzed using scanning electron microscopy (SEM) (JEOL, JSM-6510, Tokyo, Japan). Nitrogen adsorption/desorption measurements were conducted on Autosorb-IQ2 (Quantachrome, Boynton Beach, FL, USA). Brunauer–Emmett–Teller (BET) surface areas were determined over a P/P_0_ range automatically by Quadrawin. Quenched Solid State Functional Theory (QSDFT) analysis results were collected based on a carbon slit/cylindrical pore model from Quadrawin. Transmission electron microscopy (TEM) images were gathered from JEM-2010 (Tokyo, Japan). Elemental analysis was conducted using an Euro EA 3000 apparatus (Leeman Labs, Hudson, NH, USA).

The electrodes were made into slurries (active material: carbon black: Polyvinylidene fluoride (PVDF) = 6:2:2 w/w/w, NMP as solvent) and cast on Al foil with a doctor blade before drying at 100 °C in vacuum overnight. Half-cell tests with Li metal as the counter electrode in CR2032 type were adopted for electrochemical performance testing where 1 M LiPF6 in ethylene carbonate (EC) : diethyl carbonate (DEC) = 1:1 v/v was used as electrolytes, with Celgard 2400 (Celgard, Charlotte, NC, USA) as the polymer membrane separator. Galvanostatic cycle performance tests were carried out on LAND CT2001A (Wuhan LAND electronics, Wuhan, China) over 1.5~4.5V vs. Li/Li^+^. cyclic voltammetry (CV) and alternating current (AC) impedance measurements were carried out using a VMP-3 Biologic instrument (Seyssinet-Pariset, France).

## 3. Results

Pristine CTFs have a monodisperse microporous structure. Despite the fact that a microporous nature prompts a high specific surface area, the size restriction issue encountered by micropores with a size approaching that of the solvated electrolyte ion dramatically elevates the energy barrier for the solvated ion to surmount so as to facilitate the subsequent electrochemical doping reaction [36]. In addition, it has been suggested that a possible slow ion–shell desolvation process occurs during its diffusion through the micropores, which in turn severely impedes the ion transportation dynamics [37]. The end results point to the fact that a monodisperse microporous structure is unfavorable for obtaining high-rate lithium battery cathodes. To address this issue, a hierarchical micro-mesoporous structure is deemed highly effective to realize a high surface area that ensures a large number of active sites to deliver a high capacity, and at the same time maintains a high ion accessibility that translates into rapid ion transportation and excellent rate performance. Thus in this work, an in situ polymerization of a CTF on carbon materials of different dimensionalities and morphologies that serve as the nucleation sites to initiate and regulate the growth of the CTF is proposed as an effective approach to engineer their pore structure for improved electrochemical performance.

**Figure 1:**
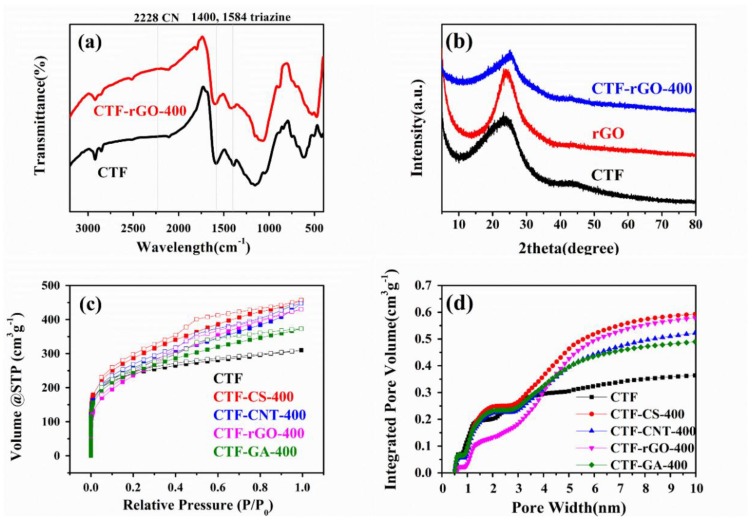
(**a**) FT-IR spectra and (**b**) X-ray diffraction (XRD) patterns of the synthesized covalent triazine-based framework (CTF) and the CTF-rGO composite; (**c**) nitrogen sorption isotherms (solid and hollow squares correspond to the adsorption and desorption process respectively); and (**d**) cumulative pore volume plot vs. pore width of CTF and its composites. rGO: reduced graphene oxide.

Taking the CTF-rGO composite as the paradigm, FT-IR is utilized to prove the successful chemical transition of the monomer. As shown in Figure 1a, the disappearance of the distinctive carbonitrile band at 2228 cm^−1^ and the concomitant appearance of absorption bands at 1400 and 1584 cm^−1^ consolidate the formation of triazine from the trimerzation of nitrile monomer. It is worth mentioning that it is CTF-1 that is synthesized in this study (henceforth the CTFs referred to in this article all refer to CTF-1, and a schematic diagram of the formation process is shown in Figure S1). Due to the inclination of the triazine-based oligomers to assemble in a 2D manner in CTFs that resembles graphene, crystalline CTF would show a similar XRD pattern to that of graphene with an eclipsed AAA structure, as suggested from MS modeling computations [20]. Accordingly, the (100) and (001) reflection peaks are located at 2θ = around 7° and 26° [20,38] for crystalline CTFs. However, due to the excessive dosage of ZnCl_2_ that obstructs periodic nucleation perpendicular to triazine planes [21,39], the crystalline order is reduced and the formation of an amorphous structure occurs, in this case featured by a broad peak around 25° and the absence of peak around 7° [40] (Figure 1b). It has been proven that a structure devoid of long range order in CTF-1 is highly beneficial to electrolyte doping and energy storage, while crystalline CTF-1 shows a very weak storage capacity [25]. Correspondingly, the CTF-rGO and composites made from other carbon materials exhibit similar and combined features to the constituent components (Figure 1b and Figure S2). 

Elemental analysis is carried out to gain information on the C/N and C/H ratios of the synthesized CTF since the values, especially the C/N ratio, are useful and versatile indicators of the CTF that determine many of its properties. For example, a low C/N ratio could suggest a large affinity of the CTF to the melted ZnCl_2_ bath that eventually affects its porosity and specific surface area [21]; and a high nitrogen content or low C/N ratio points to large CO_2_ uptake and selectivity when the CTF is used for gas capture [38,41,42]. For the CTF in this study, C/N and C/H ratios of 9.60 and 1.66 were obtained, respectively. It is suggested that the lower nitrogen and hydrogen content values than theoretically expected can be attributed to the ZnCl_2_ of a high mol equivalent adopted as in this study [38,43], which prompts nitrile cleavage or the retro-trimerization process [39,44]. Additionally, it is worth mentioning that these factors necessarily lead to structure reorganization and disruption of local order of the CTF, which are corroborated by the XRD pattern that features a broad peak around 25°, suggesting an amorphous structure. 

When growing with carbon materials of different dimensionalities, the CTF develops different pore structures that vary in substantial amounts. As assessed by the nitrogen adsorption and desorption isotherms shown in Figure 1c, the pristine CTF presents a type I isotherm, while the incorporation of carbon materials transforms the structure of the CTF. All composites exhibit type IV isotherms with associated H2-type hysteresis. The increase of the slopes and the integrated hysteresis area suggest an increase of the mesopore proportion. The pore size distribution calculated from QSDFT is shown in Figure S3. It is observed that the pristine CTF sample comes with a monodisperse microporous structure, while all the composites show more extended pore distributions at 3 nm and above. Moreover, a cumulative pore volume plot over pore width calculated from the distribution conspicuously points to the varied pore construction of different samples, where CTF-rGO-400, as compared to others, markedly exhibits suppressed micropores (<2 nm) development, while mesopores are favored during its growth (Figure 1d). By analyzing the structural shift of different composites calculated from Brunauer–Emmett–Teller (BET) and V-t (de Boer) in Table 1, it is discovered that the CTF-rGO sample delivers the most drastically reduced micropore percentage while managing to keep the large specific surface area, which is congruent with the results from Figure 1d.

**Figure 2:**
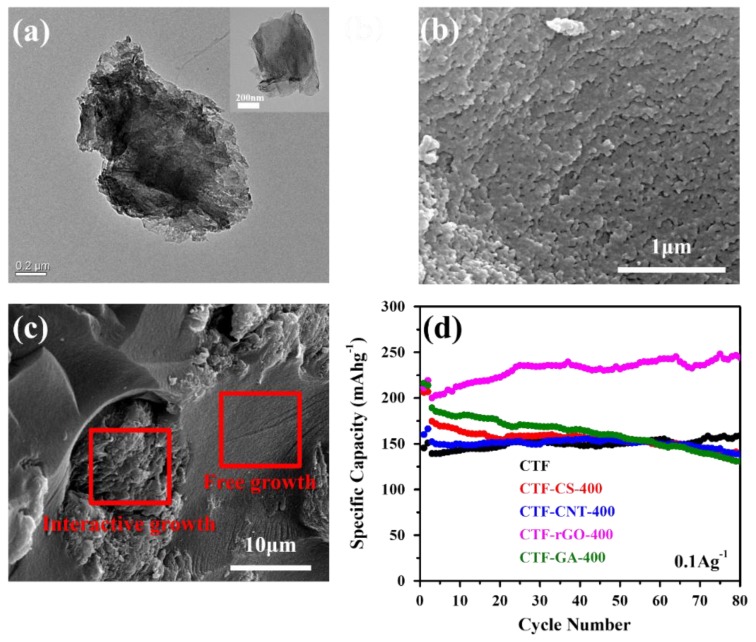
(**a**) TEM images of the CTF-rGO composite and rGO in the inset. (**b,c**) SEM images of the CTF-rGO composite and the CTF-GA composite, respectively. (**d**) The discharge capacities of CTF and all the carbon-based composites measured over 80 cycles at 0.1 A/g.

Although CS (carbon spheres) as the template could induce a huge gain in specific surface area of the composite as well as a good drop in the micropore percentage by serving as spherical nucleation sites, the very disparate morphologies and weak molecular interaction between the CTF and CS render a relatively independent growth of the CTF rather than developing an interactive core–shell structure. A similar scenario applies to the CNT-based composite. However, due to the strong inclination of the CTF to assemble in a 2D manner, which is reminiscent of graphene, plus the strong π-π interaction resulting from the conjugated polymeric structure and graphene basal plane, uniform growth of the CTF covering the surface of graphene is observed as indicated by TEM images (Figure 2a). It is worth mentioning that instead of growing into a dense layered structure on rGO, the CTF developed a fish scale-like morphology (Figure 2b) adhering tightly to the graphene surface. The rough surface feature probably results from the corrugated surface of rGO where the polymerization reaction is initiated. The unique growth manner and morphology brings radical changes to the microporous structure of the CTF and creates a large surface area along with a huge proportion of mesopores. On the other hand, GA serves as an unsatisfactory scaffold where the wild existence of numerous macropores plays invalid roles in the modification of the CTF (Figure S4). The composite shows two discernable regions where the macropores were filled with a freely grown dense CTF while the graphene flakes host a rough CTF (Figure 2c).

The electrochemical performances of all the synthesized electrodes were tested as LIB cathodes at 0.1 A/g (Figure 2d). In agreement with the established analysis, CTF-rGO with a hierarchical pore structure of the lowest proportion of micropores and large specific surface area permits readily accessible electrolytes and abundant active sites for energy storage. As a result, it delivers the highest discharge capacity among all synthesized electrodes. Moreover, the morphology compatibility as well as the strong interaction between the CTF and rGO results in an excellent cycling stability without capacity fading. Considering that CS is characteristic of a much inferior conductivity to CNTs, but the CS-based composite delivers a better performance, it is clear that structure modification rather than conductivity improvement plays a more dominant role in determining the electrochemical performance. This further consolidates the effectiveness of rGO in enhancing the electrochemical performance of the CTF.

**Figure 3:**
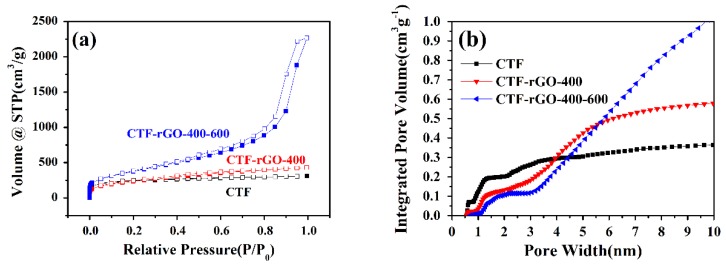
(**a**) Nitrogen sorption isotherms (solid and hollow squares correspond to the adsorption and desorption process respectively) and (**b**) the plot of cumulative pore volume vs. pore width of the CTF, CTF-rGO-400, and CTF-rGO-400-600.

As substantiated from the discussion above, it is sound to conclude that rGO fits best with the CTF when applied as a composite for the LIB cathode. To further improve its electrochemical property, pore structure engineering through thermal treatment is designed. It has been suggested that the dynamic covalent triazine-based framework is able to undergo fragmentation and reorganization under high temperatures [44]. In this regard, a combined stepwise thermal reaction where the polymerization was initiated at 400 °C for 20 h followed by 20 h at 600 °C was adopted. The new composite, denoted as CTF-rGO-400-600, also shows successful transition of the nitrile monomer to the CTF as characterized by XRD and FT-IR (Figure S5). In contrast to the CTF-rGO-400 composite obtained at 400 °C for 40 h, the higher temperature induced more complex pore structure development with more mesopores as indicated by the steep rise of the nitrogen sorption curve (Figure 3a). From QSDFT calculations, the pore width distributions for CTF-rGO-400 and CTF-rGO-400-600 were compared. Although both samples maintain micropores at around 1.2 nm, characteristic of the ring opening of CTF, CTF-rGO-400-600 shows more rigorous mesopore development at ~3 nm and above (Figure S3). Accordingly, the cumulative pore volume plot for gas adsorbed reflects the trend of further micropore reduction and mesopore construction for CTF-rGO-400-600 compared to CTF-rGO-400 (Figure 3b). The stepwise treatment results in a CTF composite with an even higher specific surface area and further reduction of the micropore percentage, as shown in Table 1. 

**Figure 4:**
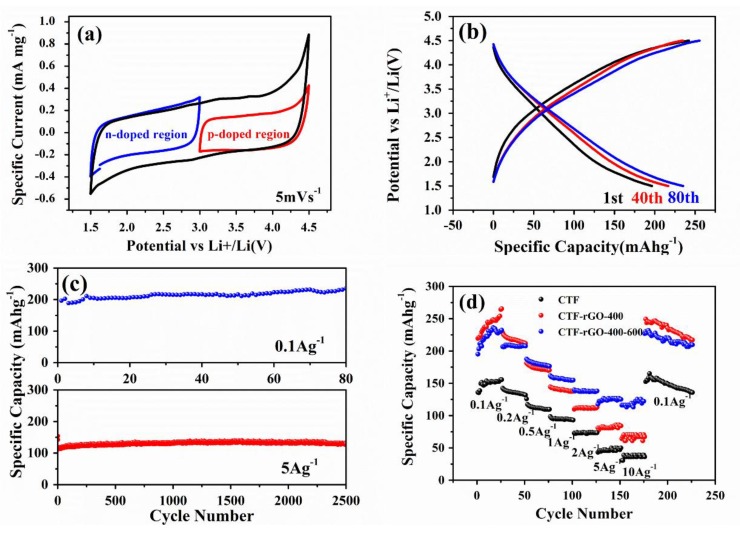
Electrochemical property tests of the CTF, CTF-rGO-400, and CTF-rGO-400-600. (**a**) CV measurement of CTF-rGO-400-600 scanned at 0.1 mV/s; (**b**) Galvanostatic charge–discharge curves of CTF-rGO-400-600 at various cycles measured at 0.1 A/g; (**c**) cycling stability test of CTF-rGO-400-600 at 0.1 A/g and at 5 A/g up to 2500 cycles; (**d**) rate performance comparison between the three samples at various current densities.

Notably, even though CTF-rGO-400-600 reshapes its pore structure at the cost of some micropores, the surface area is expanded, which is advantageous for enhanced electrochemical performance. It was reported that the CTF functions as a cathode by going through continuous bipolar redox reactions over a wide potential range, where the framework could be reversibly p-doped and n-doped [25]. When CTF-rGO-400-600 was scanned from its open circuit potential at 3 V to 4.5 V, a p doping reaction kicked in where C_3_N_3_ could be oxidized and balanced by PF_6_^−^ (Figure 4a). The opposite is expected with scanning from 4.5 V to 3 V, and the reversible reaction can be expressed as:

C_3_N_3_ + xPF_6_^−^ <=> xe^−^ + [C_3_N_3_^+x^(PF_6_^−^)_x_] (1)

In the region between 3 and 1.5 V, the n doping mechanism is ascribed to the reversible electrochemical reaction, which can simply be expressed as:

C_3_N_3_ + xe^−^ + xLi^+^ <=> [C_3_N_3_^−x^ (Li^+^)_x_] (2)

The charge–discharge curves show gradual transitions within the tested potential window, which correspond well to the continuous n/p doping mechanism. The slightly increasing capacities with cycling suggest gradual activation caused by infiltration of electrolytes into the porous matrix (Figure 4b). When cycled at a low current density of 0.1 A/g, CTF-rGO-400-600 delivers a high reversible capacity of 235 mAh/g after 80 cycles, which is outstanding when compared with the capacity of currently dominant inorganic cathodes such as LiFePO_4_, LiCoO_2_, etc. When measured at a high current density of 5A/g, CTF-rGO-400-600 still manages to contribute a remarkably stable and high capacity of 127 mAh/g after 2500 cycles (Figure 4c). As said, although micropores are regarded highly beneficial for creating an exceptionally large surface area, the ultra-small pore size approaching that of a solvated electrolyte ion hampers rapid electrochemical reactions by imposing an unfavorable barrier for ion diffusion. This effect is particularly prominent and denies fast kinetics under high-current conditions wherein electrolyte ion diffusion rather than electron transport becomes the rate-limiting step. On the other hand, relatively larger mesopores bypass the bottleneck by providing readily accessible reaction sites to accommodate solvated ions [36,37]. Thus, engineering a hierarchical micro-mesoporous structure of CTF-rGO-400-600 should be an appealing strategy to improve the sluggish dynamics of the microporous CTF, particularly at a high charge/discharge rate. CTF-rGO-400-600 shows an excellent performance, with 122 mAh/g retained even when discharged at 10 A/g. This is far superior to the performance of the pristine CTF and CTF-rGO-400. Furthermore, its large capacity can be well restored when the discharge current density falls back to 0.1 A/g, which reflects the structural stability of the composite through large currents (Figure 4d). The much improved rate performance of CTF-rGO-400-600 over the pristine CTF and CTF-rGO-400 is evidenced by electrochemical impedance spectroscopy (EIS), in which CTF-rGO-400-600 delivers a reduced charge transfer resistance at the electrode/electrolyte interface and shows a steeper linear slope in the low frequency region, suggesting a larger electrolyte ion diffusion coefficient [45] (Figure S6). 

## 4. Discussion and Conclusions

In recent years it has become a compelling idea to utilize CTFs with a large specific surface area for electrochemical energy storage applications, and the monodisperse microporous structure and low intrinsic conductivity which greatly restrict their further feasible applications should be immediately addressed. A rational design by compositing the CTF with carbon materials of different dimensionalities is herein directed towards solving the mentioned problems. Two-dimensional rGO with good morphology compatibility with the CTF as well as strong π-π intermolecular interactions with the CTF shows the greatest potential for integration with the triazine-based framework. Through this approach, CTF-rGO attains a suppressed micropore percentage by reorganization into extended mesopores while enlarging its specific surface area. This is regarded as highly advantageous for enhanced electrochemical properties. After a stepwise thermal treatment to further induce pore structure reorganization, the obtained composite delivers a large specific surface area of 1357.27 m^2^/g, a high capacity of 235 mAh/g after 80 cycles at 0.1 A/g, and outstanding stability, with 127 mAh/g retained after 2500 cycles at 5 A/g when used as an LIB cathode. The hierarchical pore structure also promotes excellent Li^+^ adsorption dynamics, with a large capacity retained at 122 mAh/g at a current density as high as 10 A/g. The discussed methodology for the production of high-capacity and high-rate organic LIB cathodes with a hierarchical pore structure might serve as an exciting solution for the currently sluggishly progressing LIB cathode industry. 

## Figures and Tables

**Table 1:** Summary and analysis of nitrogen sorption data for all synthesized CTF composites. BET: Brunauer–Emmett–Teller.

Calculation Model	BET	V-t	
	Surface Area (m^2^/g)	Micropore Area (m^2^/g)	Micropore Percentage
CTF	868.058	628.490	72.4%
CTF-CS-400	1009.801	441.733	43.7%
CTF-CNT-400	911.556	437.201	47.9%
CTF-rGO-400	1018.616	330.443	32.4%
CTF-GA-400	865.614	455.793	52.7%
CTF-rGO-400-600	1357.270	200.444	14.8%

## Supplementary Materials

The following are available online at http://www.mdpi.com/1996-1944/11/6/937/s1, Figure S1. A schematic showing the structure and formation process of CTF-1; Figure S2: XRD patterns of CTF composites with different carbon materials. (a) CTF-CS, (b) CTF-CNT; and (c) CTF-GA; Figure S3. Pore size distribution of CTF and different CTF composites calculated from QSDFT; Figure S4: An SEM image of GA showing the macroporous nature; Figure S5: The FT-IR spectra and XRD pattern of CTF-rGO-400-600 proving the formation of the polymeric framework; Figure S6: Nyquist plots derived from AC impedance measurement of CTF, CTF-rGO-400, and CTF-rGO-400-600 before cycling.
