# Supplementary material for: Rational Design of Porous Covalent Triazine-Based Framework Composites as Advanced Organic Lithium-Ion Battery Cathodes
Source: Materials (Basel). 2018 Jun 2;11(6):937. doi: 10.3390/ma11060937 (PMC6025425; doi:10.3390/ma11060937)
Supplement: Supplementary file 1 [file materials-11-00937-s001.pdf]

materials

Supplementary

Rational Design of Porous Covalent Triazine-Based Framework Composites as Advanced Organic Lithium-Ion Battery Cathodes

Ruoxin Yuan †, Wenbin Kang † and Chuhong Zhang *

State Key Laboratory of Polymer Materials Engineering, Polymer Research Institute of Sichuan University, Chengdu 610065, China; 2015223090029@stu.scu.edu.cn (R.Y.); wenbin.kang@scu.edu.cn (W.K.)

* Correspondence: chuhong.zhang@scu.edu.cn

† These authors contribute equally to this work.

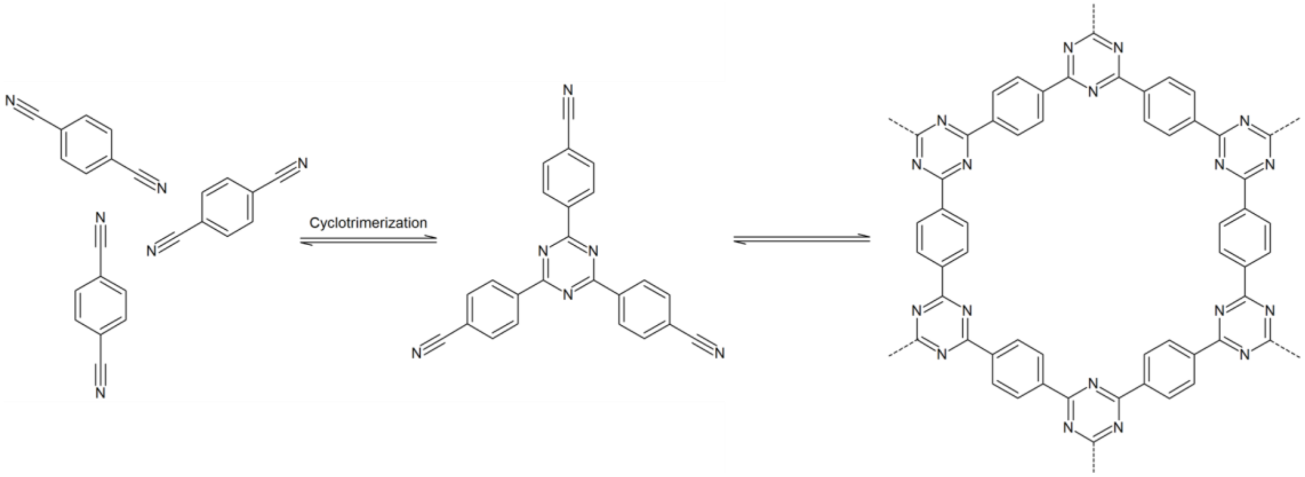

Figure S1. A schematic showing the structure and formation process of CTF-1.

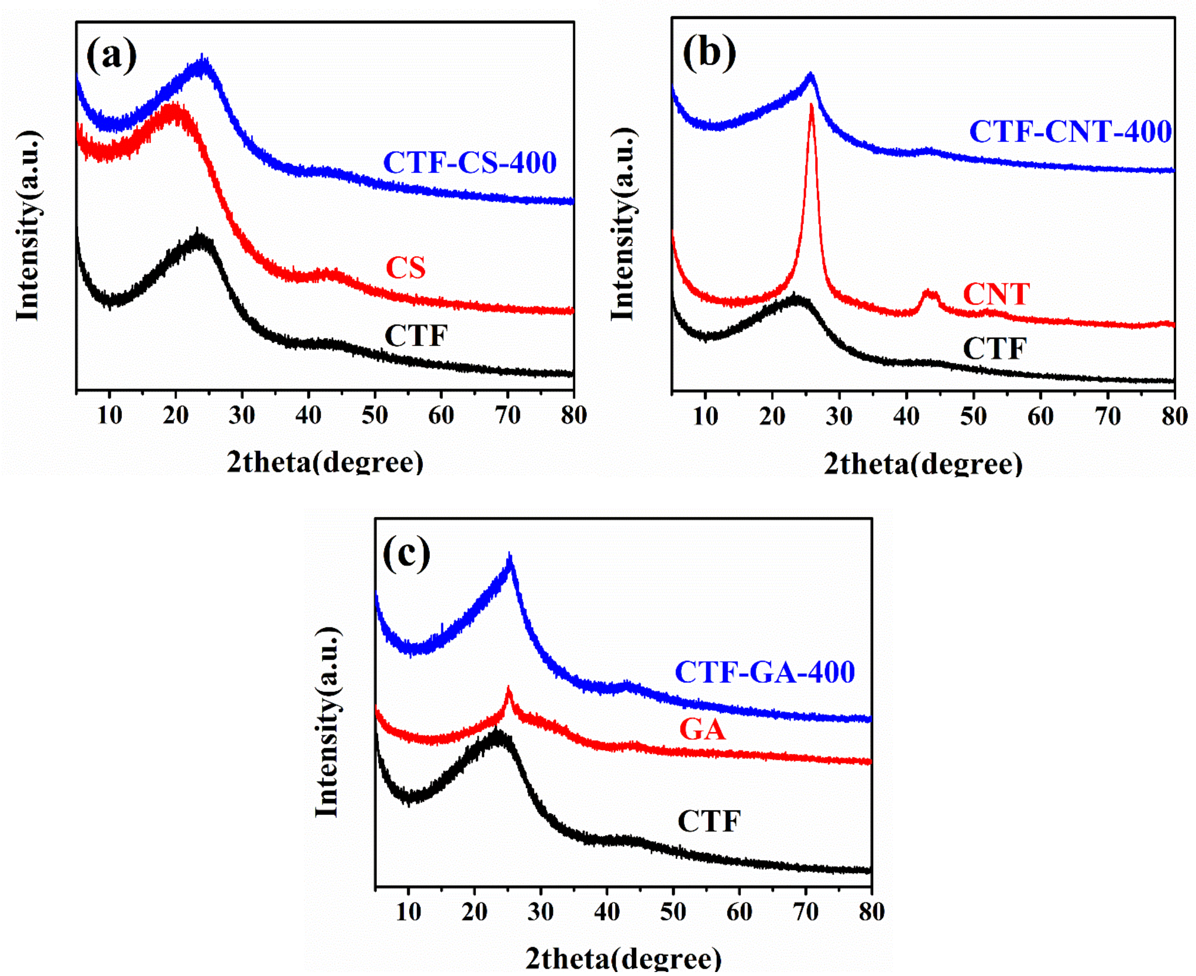

Figure S2. XRD patterns of CTF composites with different carbon materials. a) CTF-CS, b) CTF- CNT and c) CTF-GA.

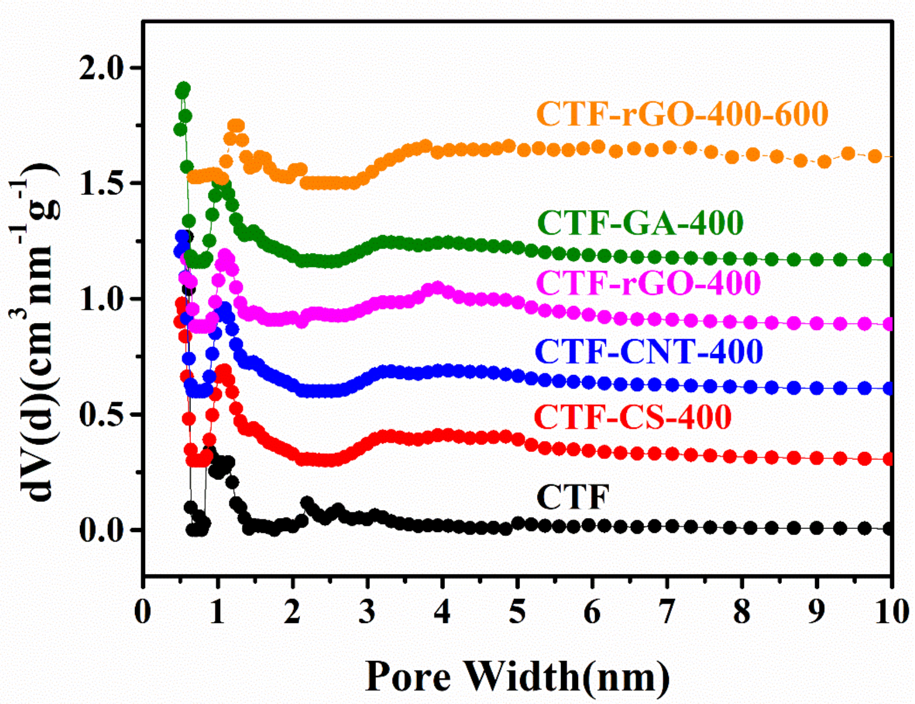

Figure S3. Pore size distribution of CTF and different CTF composites calculated from QSDFT.

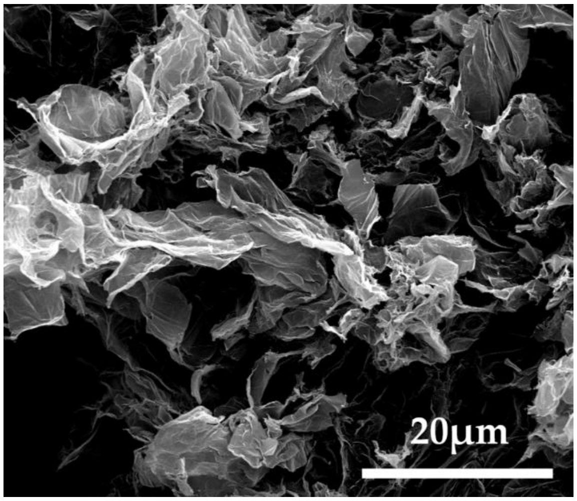

Figure S4. An SEM image of GA showing the macroporous nature.

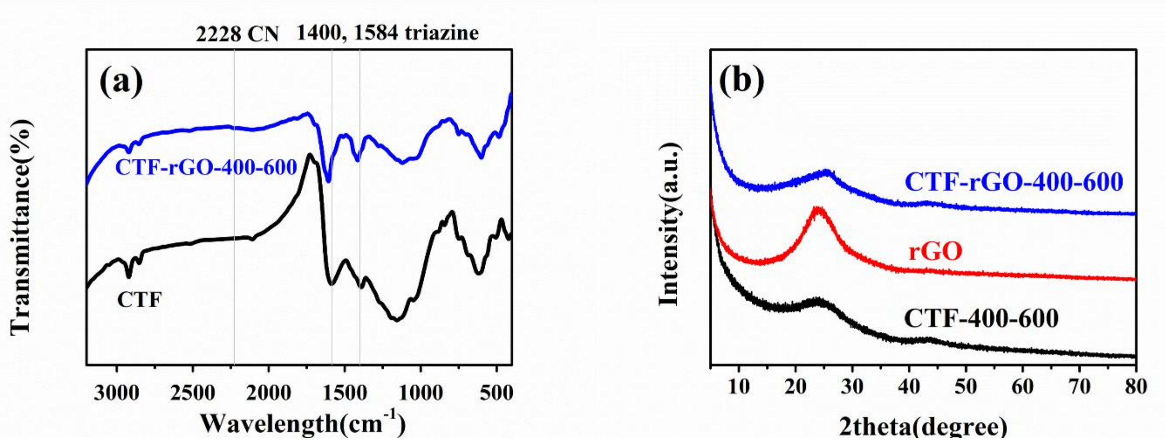

Figure S5. The FT-IR spectra and XRD pattern of CTF-rGO-400-600 proving the formation of the polymeric framework.

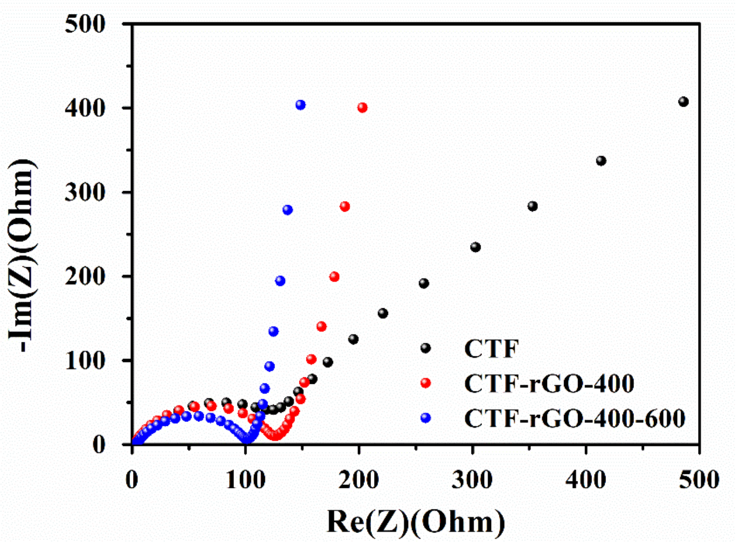

Figure S6. Nyquist plots derived from AC impedance measurement of CTF, CTF-rGO-400 and CTF-rGO-400-600 before cycling.
